# Estimation of cell response in fractionation radiotherapy using different methods derived from linear quadratic model

**DOI:** 10.1515/raon-2015-0040

**Published:** 2015-11-27

**Authors:** Safoora Nikzad, Bijan Hashemi, Golshan Mahmoudi, Milad Baradaran-Ghahfarokhi

**Affiliations:** 1 Department of Medical Physics, Faculty of Medicine, Hamadan University of Medical Sciences, Hamadan, Iran; 2 Department of Medical Physics, Tarbiat Modares University, Tehran, Iran; 3 Department of Medical Physics, School of Medicine, Sabzevar University of Medical Sciences, Sabzevar, Iran; 4 Department of Medical Physics and Medical Engineering & Medical Student’s Research Center, School of Medicine, Isfahan University of Medical Sciences, Isfahan, Iran; 5 Department of Medical Radiation Engineering, Faculty of Advanced Sciences & Technologies, Isfahan University, Isfahan, Iran

**Keywords:** fractionation radiotherapy, survival, dose per fraction, number of fractions, linear quadratic model

## Abstract

**Background:**

The aim of this study was to use various theoretical methods derived from the Linear Quadratic (LQ) model to calculate the effects of number of subfractions, time intervals between subfractions, dose per subfraction, and overall fraction time on the cells’ survival. Comparison of the results with experimental outcomes of melanoma and breast adenocarcinoma cells was also performed. Finally, the best matched method with experimental outcomes is introduced as the most accurate method in predicting the cell response.

**Materials and methods.:**

The most widely used theoretical methods in the literature, presented by Keall *et al*., Brenner, and Mu *et al*., were used to calculate the cells’ survival following radiotherapy with different treatment schemes. The overall treatment times were ranged from 15 to 240 minutes. To investigate the effects of number of subfractions and dose per subfraction, the cells’ survival after different treatment delivery scenarios were calculated through fixed overall treatment times of 30, 60 and 240 minutes. The experimental tests were done for dose of 4 Gy. The results were compared with those of the theoretical outcomes.

**Results:**

The most affective parameter on the cells’ survival was the overall treatment time. However, the number of subfractions per fractions was another effecting parameter in the theoretical models. This parameter showed no significant effect on the cells’ survival in experimental schemes. The variations in number of subfractions per each fraction showed different results on the cells’ survival, calculated by Keall *et al*. and Brenner methods (P<0.05).

**Conclusions:**

Mu *et al*. method can predict the cells’ survival following fractionation radiotherapy more accurately than the other models. Using Mu *et al*. method, as an accurate and simple method to predict the cell response after fractionation radiotherapy, is suggested for clinical applications.

## Introduction

Radiotherapy is one of the main procedures of cancer treatment. The goal of radiotherapy is to deliver as much dose to the tumor site while keeping the dose to the surrounding normal tissues as low as possible.^1^, ^2^ In radiotherapy, in addition to the conventional techniques used in clinical practice, some state of the art specialized techniques such as Intensity Modulated Radiation Therapy (IMRT), Respiratory-Gated, stereotactic, and Image Guide Radiotherapy (IGRT) have also been developed.^3^–^7^ These modern techniques optimize the radiotherapy dose distribution since they include more segments in the radiation field which are usually shaped using more complicated equipment.^3^–^7^ These techniques enhance tumor local control and have lower radiation-induced toxicities in normal organs around the tumor compared to conventional techniques. Moreover, they vary in the dose delivery due to using more subfractions per each treatment fraction, different treatment times between subfractions, and the prolonged treatment time of one fraction.^4^,^8^–^15^

The radiobiological efficiency of these techniques might be different from conventional one mainly due to the repair of sublethal damages.^8^–^16^ However, the rate and the mechanism of repair is a complicated function of different parameters such as dose per fraction, dose rate, repairs half time, and state and nature of the organs of interest (*i.e*. α/β ratio of the organ).^8^–^16^

To predict the results of different radiation delivery procedures on the cells’ survival, the basic theoretical model is the incomplete repair model of Thames^17^ generalized to multiple fractions by Nilsson *et al*.^18^ that is a developed form of Linear Quadratic (LQ) model. Some studies have investigated the effects of prolonged time of radiation delivery on the survival of some cell lines and compared the results with theoretical methods derived from the LQ model.^8^–^15^

Although these theoretical methods are all derived from the basic LQ model, however, the rate of agreement between their results in researches and experiments was significantly different for diverse dose schedules.^8^,^9^,^19^–^21^

Therefore, more investigations are needed in order to evaluate the effect of various treatment factors on the cells’ survival. In addition, it seems beneficial to compare the results of these methods theoretically and experimentally in order to find the best method that can be used to predict the cells’ survival after different fractionation radiotherapy schemes.

The aim of this study was to compare various theoretical methods widely used in the literature^8^,^9^,^19^–^21^ to estimate the effects of number of subfractions, time intervals between subfractions, dose per subfraction, and overall fraction time on the F10B16 skin melanoma and 4T1 breast adenocarcinoma cells’ survival. Comparison of the results with experimental outcomes of melanoma and breast adenocarcinoma cells was also performed. Moreover, in this work, the best matched method with experimental outcomes is introduced as the most accurate one in predicting the cell response in fractionation radiotherapy.

## Materials and methods

### Theoretical methods

Three methods of calculation derived from LQ model, presented by Keall *et al*., Brenner, and Mu *et al*.^8^,^9^,^19^–^21^, were compared to investigate the effect of different dose schemes (dose per subfraction, time intervals between subfractions, total treatment time of each fraction) on the survival of F10B16 skin melanoma and 4T1 breast adenocarcinoma cells. The basic idea of these methods is based on the completed LQ model as:

[1] $$S=\text{exp}(-\alpha D-G\beta D^{2})$$

Which is a developed form of the basic LQ model:

[2] $$S=\text{exp}(-\alpha D-\beta D^{2})$$

Where α and β are cell parameters, D is the total dose delivered to the cells, S is the survival fraction of cells, and G parameter is defined in intermittent radiotherapy to investigate the effect of subfractions. The G parameter has been formulated differently by various investigators.^8^,^9^,^19^–^21^

The first method (method I) was presented by Keall *et al*.^9^ They have experimentally and theoretically investigated the temporal effects of respiratory-gated and IMRT treatment delivery for dose of 2 Gy and in the total treatment times of 1.67 min (in conformal radiotherapy) to 15 min (in gated IMRT) on the cells’ survival. Keall *et al*. have used a simplified form of G to predict the cells’ survival and have compared the outcomes with experimental results.^9^ They have assumed negligible cell proliferation and unchanging radiosensitivity.^9^ According to Keall *et al*. study, the G parameter is calculated as^9^:

[3] $$\text{G}=\frac{1}{\text{n}}\left[2\left(\frac{\mu \tau -1+\text{exp}(-\mu \tau )}{{\left(\mu \tau \right)}^{2}}\right)+2\left(\frac{\text{cosh}(\mu \tau )-1}{{\left(\mu \tau \right)}^{2}}\right)\times (\frac{2}{\text{n}}\left(\frac{\emptyset }{1-\emptyset }\right)\left(\text{n}-\frac{1-\emptyset^{\text{n}}}{1-\emptyset }\right))\right][3]$$

Where

[4] ∅=exp(−μ(τ+Δt))

In this method, μ is the rate constant for repair of sublethal damages, n is the number of subfractions, τ is the time of exposure and Δt is the time between subfractions. This method assumes a constant value for both exposure time (t) and the time between exposures (Δt).^9^ Keall *et al*. results showed no significant difference between the experimental observations and theoretical calculations.^9^ Moreover, this method indicated a good agreement with experimental results for the total dose of 2 Gy.^9^

The second method (method II) was utilized by Brenner.^20^ This method was also proposed in some review papers.^19^,^21^ Brenner has simplified the LQ model and experimentally and theoretically investigated the temporal effects of fractionation treatment delivery on *in vitro* survival.^20^

In Brenner method, the G factor accounts for fraction protection and acts on the quadratic component as follow^20^:

[5] $$G=\frac{2}{\tau^{2}}\int_{0}^{\tau }\int_{0}^{t}e^{-\mu (t-t^{\prime })}dt^{\prime }dt=\frac{2}{{\left(\mu \tau \right)}^{2}}(\mu \tau +\text{exp}(-\tau \tau )-1)$$

In this method, the used parameters are the same as Keall *et al*. method.^9^ As this formula (equation 5) shows, the effects of time intervals between subfractions are ignored, however, Brenner has confirmed that there was a good agreement between the outcomes of this formula and the experimental results.^20^ Therefore, it has been proposed that, this formula can be used to calculate the cell response after prolonged treatment delivery.^20^ In addition, this method can be employed to calculate the protraction effects in a single fractionation delivered at a constant rate, splitting dose, multi-fraction irradiation protocols and continuous low dose rates radiotherapy such as brachytherapy.^20^

The third theoretical method (method III) was reported by Mu *et al*.^8^ In Mu *et al*. study, the G parameter is defined as below^8^:

[6] $$G=\frac{2}{n^{2}}\left[\frac{\text{exp}(-\mu \Delta t)}{1-\text{exp}(-\mu \Delta t)}\right]\left[n-\frac{1-{\left(\text{exp}(-\mu \Delta t)\right)}^{n}}{1-\text{exp}(-\mu \Delta t)}\right]+\frac{1}{n}$$

All the used parameters in this method are explained above. In this method, it is assumed that there is no recovery during actual irradiations but rather during the time between subfactions.^8^

### Cell culture and assay

The cells were cultured in plastic flasks at 37°C in a humidified atmosphere of 50 mL/L CO2 and 95% air with the RPMI1640 medium containing 10–15% fetal calf serum (FCS or FBS) with 100 U/mL penicillin and 100 μg/mL streptomycin.

Due to tree shaped structure of these cell lines, complexity of counting their colonies, and significant number of samples used in this study, an automated and faster assay method was used. Therefore, instead of the clonogenic assay, the multi 3-(4, 5-dimethylthiazol-2-yl)-2, 5-diphenyltetrazoliumbromide (MTT) assay was used. This method was offered in other similar researches^22^–^24^ and all the details of experimental procedure are published in papers by our team for these two cell lines (F10B16 melanoma and 4T1 breast adenocarcinoma) of interest.^25^,^26^

### Theoretical schemes

In this paper, α and β parameters were calculated using the basic LQ model (equation 2). Hence, the cell survival fractions (S) following doses of 2, 4, 6, 8 and 10 Gy were experimentally determined for both melanoma and breast adenocarcinoma cells and then inserted in the basic formula of the LQ model. Using the S and D parameters and inserting them in the mentioned formula, the survival curves of cell lines were drawn and α and β parameters were derived using the MATLAB software (Version 7.11, R2010b, MathWorks, USA).^8^,^25^

In order to determine the time constant for repair of sublethal damage (T_1/2_), the cells were exposed in two fractions with different time intervals between the fractions. Then, the surviving fraction was plotted against the time between fractions and finally the half value of sublethal damage repair was investigated.^8^,^25^

All the cell’s parameters for both cell lines of interest, used in this study, are illustrated in Table 1.

Different treatment schemes were designed in order to investigate the effects of the most important radiobiological parameters including; the number of subfractions, time intervals between subfractions, subfraction doses, and overall treatment time, in complex radiotherapy practices.

To investigate the effect of total treatment time, the survival fraction (SF) were calculated for dose of 2, 4 and 6 Gy in two subfractions of 1, 2 and 3 Gy, respectively. The overall treatment times were ranged from 15 to 240 minutes. Although, total treatment time in complicated radiotherapy is about 1 hour and longer treatment time is not practical, however, we followed the investigations for up to 4 hours to determine comprehensive results and investigate the ability of the developed models to predict the cells’ survival.

To investigate the effects of increasing the number of subfractions and dose per subfraction, the survival was calculated for total dose of 2, 4 and 6 Gy in 4 and 8 subfractions as follow: 4 fractions of 0.5 Gy and 8 fractions of 0.25 Gy (both for a total dose of 2 Gy), 4 fractions of 1 Gy and 8 fractions of 0.5 Gy (both for a total dose of 4 Gy) and 4 fractions of 1.25 Gy and 8 fractions of 0.75 Gy (both for a total dose of 6 Gy). They all delivered through fixed overall treatment times of 30, 60 and 240 minutes.

It should be noted that the theoretical methods presented by Keall *et al*., Brenner, and Mu *et al*. can be used in predicting survival in fractionation radiotherapy and some of them have flaw in predicting survival when the dose is delivered continuously in one fraction.^8^,^9^ Therefore, in this work, the basic LQ model (equation 2) was used to predict the cell survival following continuous dose delivery.

### Experimental schemes

The cells were picked out from the flasks when they reached to linear phase of exponential grow in the day before irradiation and were put in 96 well plates with density of 1000 cells in each well.^22^–^25^ There were 7 samples for each experiment and, to avoid the variability inherent to the assay used, all tests were performed for 3 independent experiments. A Co-60 source with a dose rate of 0.81 Gy/min was used for irradiation. The ionizing radiation was delivered in a 25×25 cm^2^ field size. All irradiations were performed at a distance of 20 cm between the radiation sources and plate.

To measure the absorbed dose rate of the Cobalt-60 beam, a Farmer-type ionization chamber with a standard ^60^Co buildup cap, and positioned in air using a customized stand, was used. “For traceability to international standards, the ionization chamber was calibrated in comparison with the response of the Secondary Standard Dosimetry Laboratory (SSDL, Karaj Complex, Atomic Energy Organization of Iran) reference and working standard ionization chambers in the ^60^Co gamma ray beam of a teletherapy unit. All of the SSDL ionization chambers used for calibrations are themselves calibrated at the International Atomic Energy Agency (IAEA) dosimetry laboratory”.^27^

To design the experimental tests, firstly, continuous radiation with doses of 2, 4 and 6 Gy, similar to conventional radiotherapy techniques, were delivered to the cells. Next, to investigate the effect of overall treatment time on the cells’ survival, the same as the theoretical schedules, 6 groups from both of the studied cell lines were exposed to 2, 4 and 6 Gy in two subfractions with dose of 1, 2 and 3 Gy, respectively. In this step, the overall treatment time was 15 to 240 minutes. Then, to simulate the effects of the number of subfractions as well as dose per subfraction, 4 and 8 subfractions with dose of 1 and 0.5 Gy, respectively (total dose of 4 Gy), were delivered to the cells at overall treatment times of 30, 60 and 240 minute. After that, the results were compared with those of continuous radiation.

**FIGURE 1.**
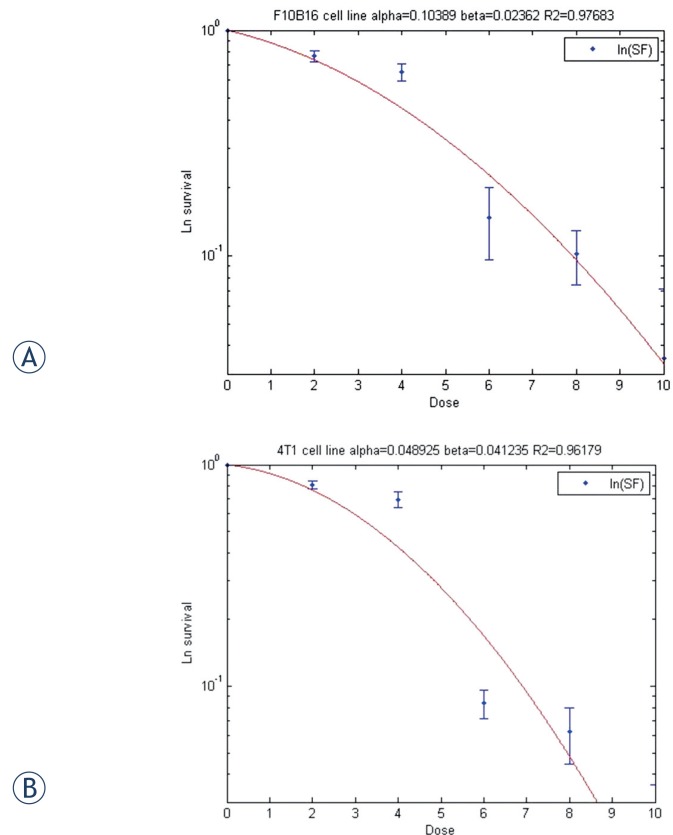
The survival curves of the **(A)** melanoma F10B16 and **(B)** breast adenocarcinoma 4T1 cell lines (R2 is a statistical measure of how close the data are to the fitted regression curve. It is also known as the coefficient of determination).

It should be noted that, although the conventional treatment dose used in clinical situation is approximately 2 Gy per fraction^8^,^9^, however, the effect of this low level of dose on the cell culture environment was negligible for the two cell lines of interest (Figure 1), and consequently the dose of 4 Gy was used in this experiment.

## Statistical analysis

Statistical analysis was performed using the SPSS software version 14 (SPSS, Inc., Chicago, IL, USA). To assess the effects of different irradiation protocols, the analysis of variance (ANOVA) was used. A significant level of 0.05 was considered to the tests.

## Results

**FIGURE 2.**
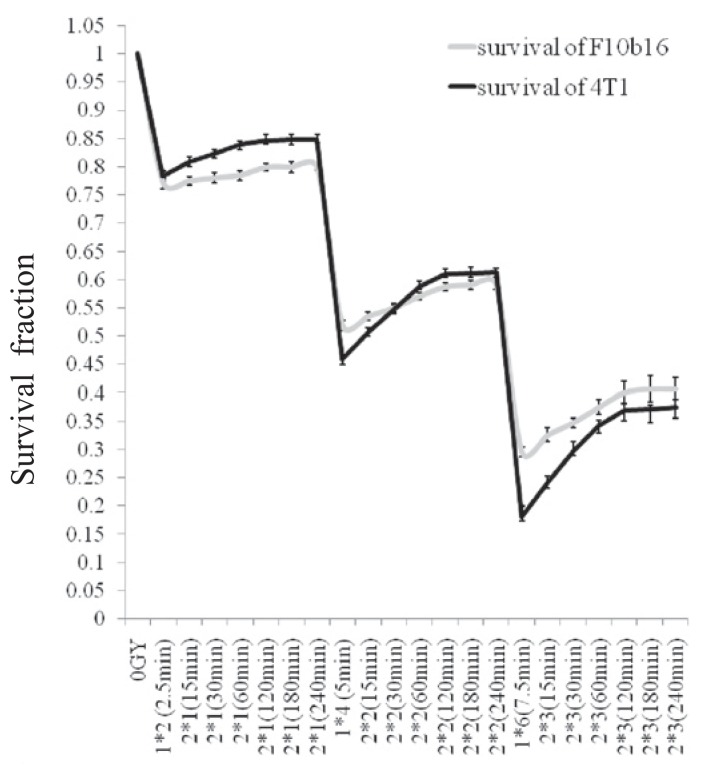
The survival fraction of F10B16 and 4T1 cells in dose levels of 2, 4 and 6 Gy delivered continuously and in two subfractions of 1, 2 and 3 Gy, respectively, for overall treatment time of 15 to 240 minutes.

**FIGURE 3.**
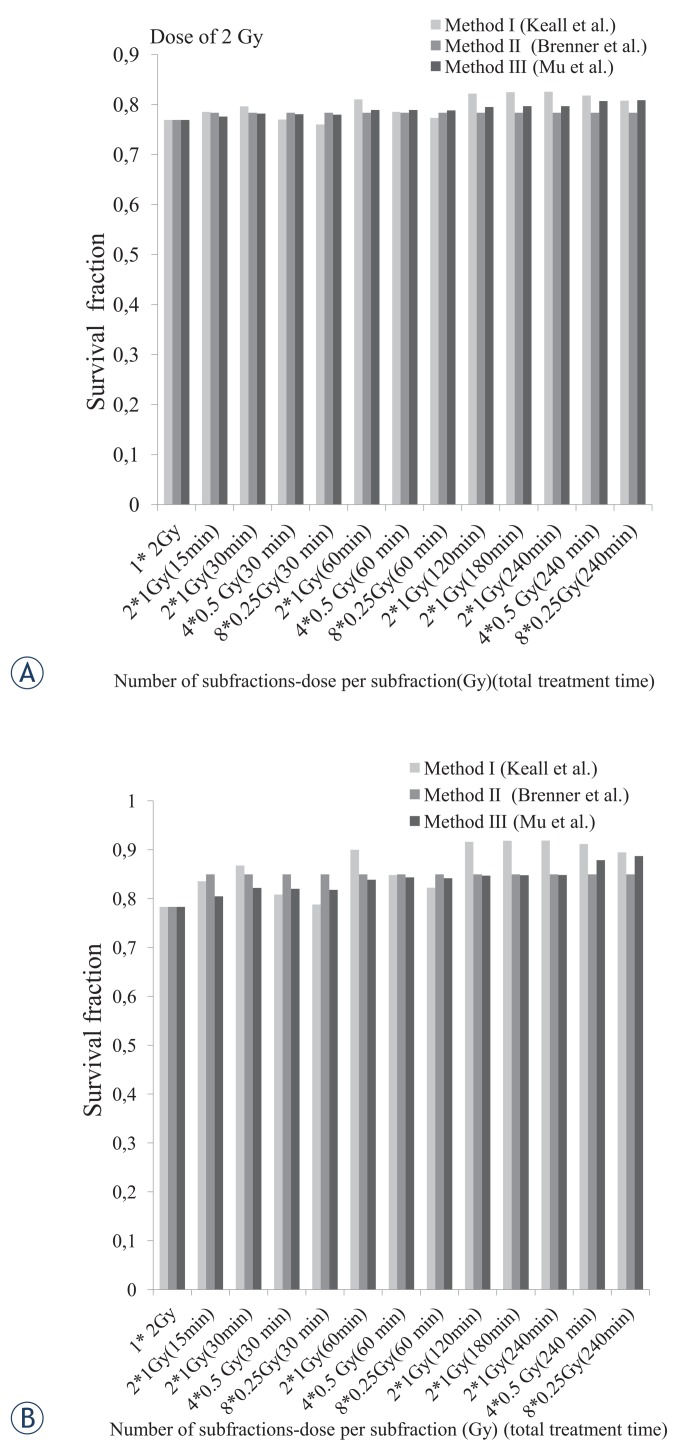
The survival fraction predicted by the used methods for F10B16 melanoma **(A)** and 4T1 breast adenocarcinoma **(B**) cell lines in different fraction numbers, dose per fractions, and total treatment times, for dose of 2 Gy.

**FIGURE 4.**
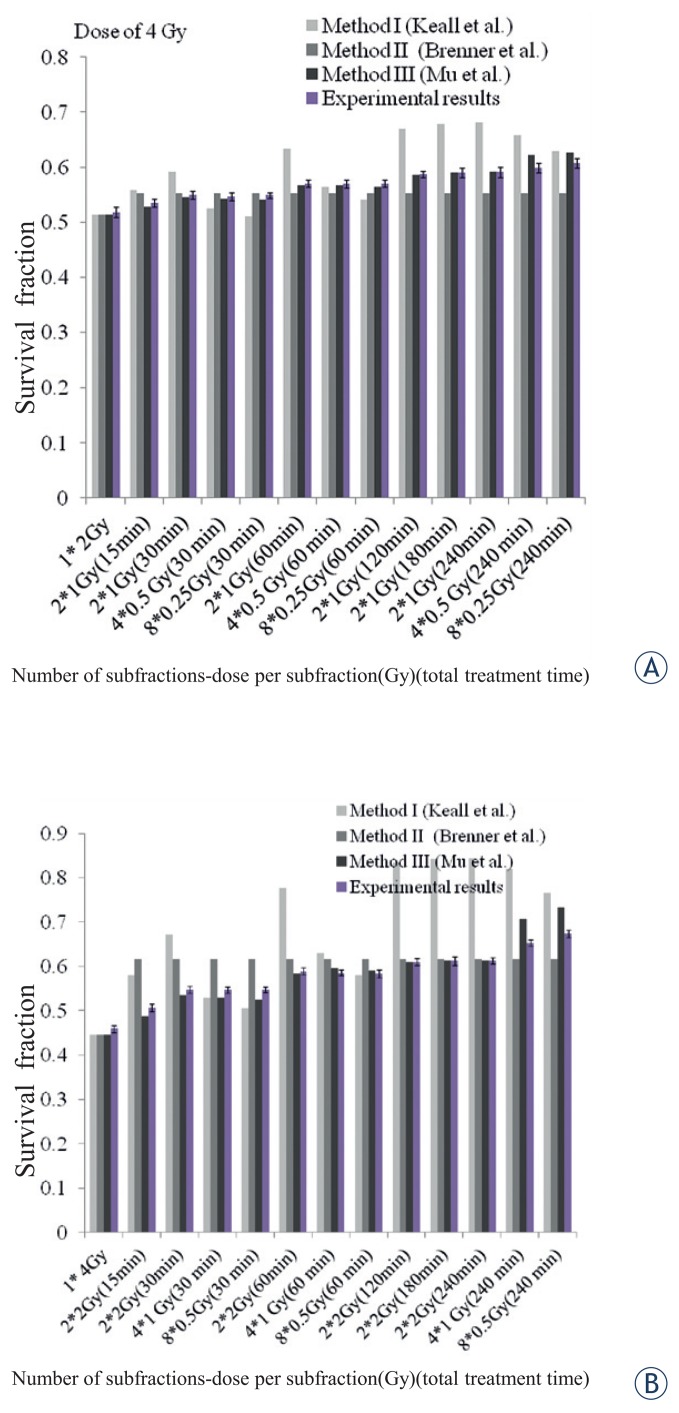
The survival fraction predicted by the used methods as well as the experimental results for F10B16 melanoma **(A)** and 4T1 breast adenocarcinoma **(B)** cell lines in different fraction numbers, dose per fractions, and total treatment times, for dose of 4 Gy.

**FIGURE 5.**
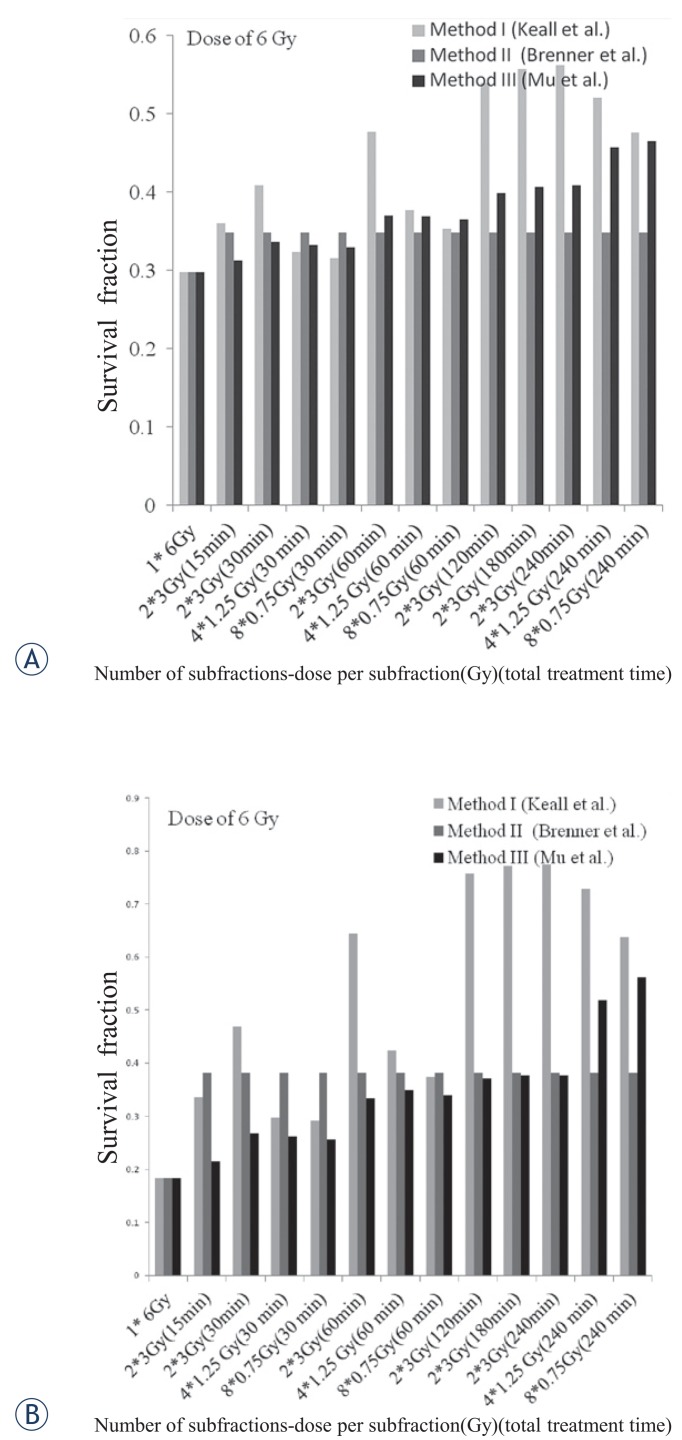
The survival fraction predicted by the used methods for F10B16 melanoma **(A)** and 4T1 breast adenocarcinoma **(B)** cell lines in different fraction numbers, dose per fractions, and total treatment times, for dose of 6 Gy.

Figure 1 illustrates the survival curves for the two cells of interest as well as the calculated α and β parameters. Figures 2, gives the survival of the cells in continuous radiation with dose of 2, 4 and 6 Gy and also in fractionation delivery in two subfractions of 1, 2 and 3 Gy, during the overall treatment time of 15 to 240 minute. Figures 3 to 5, show the predicted survival using the theoretical methods of Keall *et al.*, Brenner, and Mu *et al.*, as well as the experimental results. For a total dose of 2 Gy and all irradiation times in both two cell lines of interest (4T1 and F10B16), there was no significant difference (P<0.05) between the calculated survival by the three used methods (Figure 3).

For a dose of 4 Gy, there was no significant difference (P<0.05) between survivals calculated by three methods in total treatment time of up to 60 minute (Figure 4). After 60 minute, for the F1B16 cells, there was a significant difference between method I and the other two methods. While in the treatment time less than 240 minute there was no significant difference between methods I and III (P<0.05). For the 4T1 cells, there were significant differences (P<0.05) in calculated survival between method I and the two other methods. Considering total treatment time, these variations increased considerably after 60 minute. Small differences observed between methods II and III in groups with 4 or 8 subfractions (Figure 4).

For a total dose of 6 Gy, the results of calculations for the F10B16 cells were the same as dose of 4 Gy (Figure 5). For the 4T1 cells, a significant difference between the used methods observed, especially between the method I and the other two methods (Figure 5).

These results showed that, when the total treatment time increased, the survival of both two cell lines increased significantly according to all three methods.

Increasing the number of subfractions showed different results according to the used methods.

According to the method I, increasing the number of subfractions in a fixed total treatment time reduced the survival in all three doses of 2, 4 and 6 Gy. The predicted survival according to the method II did not show any significant difference (P<0.05) due to the variations in number of subfractions.

The calculated survival by the method III for F10B16 melanoma cell line, showed a significant decrease by increasing the number of subfractions from 2 to 4 and 4 to 8, for total dose of 2, 4 and 6 Gy and both treatment times of 30 and 60 minute. While, for the 240 minute treatment time, increasing the number of subfractions increased the survival of the cells (Figures 3 to 5).

For 4T1 cell line, increasing the number of subfractions decreased the survival of the cells in 30 minute treatment time. For the total treatment time of 60 minute, increasing the number of subfractions from 2 to 4 fractions enhanced the survival of the cells, whereas, increasing the subfractions to 8 declined the cells survival. For the 240 minute treatment time, increasing the number of subfractions raised the cells survival (Figures 3 to 5).

Considering the three used methods, differences between the exposed groups to 2 Gy was not significant for the F10B16 cells and was negligible for 4T1 cells. Therefore, to investigate the effects of number of subfractions and dose per subfraction in experimental investigations, a total dose of 4 Gy was used. The results of this experiment were assessed in 2, 4 and 8 fractions during treatment times of 30, 60 and 240 min, for both F10B16 and 4T1 cell lines.

Comparisons between the experimental results with those calculated by the three used methods showed that experimental results were in a good agreement with method III. The results of experimental investigations and the calculated survival by the method III are shown in Table 2.

The results showed that, in a fixed overall treatment time, there was no statistical significant difference (P<0.05) between the irradiated groups in different subfractions. Considering the overall treatment time, there was an agreement between experimental results and those predicted by the method III for the irradiated cells in total treatment time of 1 h, as opposed to the 4 h irradiated group.

## Discussion

Recently, some researchers have shown the effect of prolonged dose delivery time on the cell survivals.^8^–^15^ In this regard, several models have been offered to predict the effects of variations in the treatment procedures on the cells survival.^8^,^9^,^20^ One of these models is the developed LQ model by Thames and Dale.^17^ However, different theoretical methods have been derived from this model in some researches in order to predict the survival of cells after prolonged dose delivery schemes.^8^,^9^,^10^,^19^ As stated earlier, these researches have just investigated the effect of total treatment time and have not considered the effects of number of subfractions, dose per subfarction and the time intervals between subfractions in detail.

Therefore, more investigations were needed in order to determine the effect of different treatment factors on cells’ survival. In addition, it seems useful to compare the results of these methods theoretically and experimentally in order to find the best method for clinical application in fractionation radiotherapy.

In this study, three calculation methods derived from the basic LQ model proposed in different researches^8^,^9^,^20^ were used to evaluate the effects of different parameters such as total treatment time, number of subfractions, and subfractions interval on survival of cell lines with constant α, β and μ parameters. Then, the results were compared with experimental outcomes of F10B16 skin melanoma and 4T1 breast adenocarcinoma cells.

Comparison between the results of the used three methods with those of experimental results showed that method III (Mu *et al*. model) was in a better agreement with experimental outcomes. Mu *et al*. proposed a method to calculate the effect of prolonged treatment time on the Chinese hamster fibroblasts (V79-379-A) cells’ survival for total treatment dose of 2 and 8 Gy. They have shown that, there is a good agreement between experimental and theoretical results for the total dose of 2 Gy and treatment time below 1 hour. While in our study, different mathematical methods presented by Keall *et al*., Brenner and Mu *et al*., were used to calculate the cells’ survival after different treatment schemes such as 2, 4, and 6 Gy continuous dose in two subfractions with dose of 1, 2, and 3 Gy, respectively. In this work, to investigate the effects of the number of subfractions and dose per subfraction, the cells’ survival after total doses of 2 Gy (4 subfractions of 0. 5 Gy and 8 subfractions of 0.25 Gy), 4 Gy (4 subfractions of 1 Gy and 8 subfractions of 0.5 Gy), 6 Gy (4 subfractions of 1.25 Gy and 8 subfractions of 0.75 Gy) were calculated through fixed overall treatment times of 30, 60 and 240 minutes.

Considering the method III investigations in predicting the F10B16 cells survival (T1/2 = 30 minute), it is expected that increasing the number of subfractions reduced the survival, in total treatment times of 30 and 60 minute. The reason was due to the repair of sublethal damages. For all defined subfractions (2, 4 and 8), the intervals between subfractions was lower than T1/2, therefore, after the first irradiation there was not enough time for the cells to repair their sublethal damages, hence the survival reduced. This effect was found for 4T1 cells in total treatment time of 30 minute. However, for the 60 minute treatment time, considering the T1/2 of about 20 minute (significantly lower than F10B16) the time between 4 subfractions was higher than the repair time, and therefore, after irradiation in the first subfraction the damages were repaired before starting the next exposure, consequently the survival increased. However, for the 8 subfractions in 60 minute treatment time, the results were the same as before. These explanations can justify the behavior of the used both two cell lines in 240 minute treatment time, too. Therefore, the survival of cells increased for this total treatment time.

Experimental results showed that increasing the total treatment time, similarly occurred in new complicated methods such as IMRT, increased the cell survival in both cell lines and all three total dose of 2, 4 and 6 Gy in up to 2 hour treatment time. However, the extent of this effect was not considerable for F10B16 cells with shorter T1/2, and was negligible for the dose of 2 Gy for this cell line.

Moreover, the results of this research confirmed that a cell with lower α/β ratio is considered to have a greater ability to undergo sublethal damage repair. The rate of sublethal damage repair may be represented by T1/2; therefore, cells with a shorter T1/2 have more repairs. In addition, the survival of 4T1 cell line with lower α/β and T1/2 was dramatically different than the F10B16, when the time interval between subfractions increased.

In total treatment time of 4 hours, both theoretical and experimental results showed an increase in survival with fractionated irradiation.

Some studies have been performed to investigate the ability of LQ model in predicting the survival in low dose levels (< 1 Gy).^21^,^28^,^29^ Cherubini *et al*. and Jones *et al*. explained that in low doses (less than 1 Gy) the LQ model cannot predict the cell survival accurately.^28^,^21^ While, Smith *et al*. claimed that the LQ model calculates the survival precisely in such low doses.^29^ Brenner has shown that in total dose of 2 to 15 Gy, the LQ model can accurately predict the survival in *in-vitro* and *in-vivo* conditions.20 In this study, in line with Brenner, the results suggest that, in fractionation radiotherapy, the developed LQ model can potentially reach close agreement with reality in total treatment dose of 2 to 4 Gy.

Compared with other studies, using small subfractions of 0.25–0.5 Gy, Marples *et al*.^30^, and Mu *et al*.^8^ investigated the phenomenon of hypersensitivity to low doses per fraction. Marples *et al.* showed that, this would lead to a more effective cell killing than predicated by the LQ model.^30^ While Mu *et al*. study showed that there was no evidence for such effect since it should have resulted in lower survival than expected and not higher.^8^ They explained that this effect is perhaps because of the effective dose rate in each fraction which is too high to avoid activating a possible repair.^8^ However, in our study which lower dose rate was used, cell killing reached close agreement to the amount predicted by the LQ model that is in an agreement with the Marples *et al*. result. The factors that influence the dose rate are radical recombination and sublethal damage repair.^30^ It should be noted that, at the dose levels and dose rates encountered in radiotherapy, the effect of radical recombination on cell killing is negligible.^31^ Ling *et al*.^32^ and Michaels *et al*.^33^ have compared the survival of CHO cells at dose rates of 0.6 Gy/min from a Co-60 unit, and their results showed that the obtained survival curves were exactly the same with up to 15 Gy/min dose rates. Hence, based on the results of our study and comparisons with other works, an idea to reduce the effect of fractionation or prolonged treatment time is using higher dose rates or more treatment dose in one fraction.

In other work by Keall *et al*.^9^, they have shown that both respiratory gating and IMRT delivery will decrease survival compared with continuous delivery of the same dose in the same overall time. Therefore, for a given treatment time, delivery method is another factor affecting the cell survival.

## Conclusions

According to presented experimental and theoretical results, in treatment of tumors in radiotherapy by new complicated methods, this should be noted that exceeding the treatment time will increase the survival of tumor cells and may decrease tumor control. Increasing the number of subfractions in a course of treatment could reduce the cell survivals if the fractions time interval be lower than the repair time of sublethal damages. Although, this parameter has a negligible effect on the survival of the cell lines of interest in our experimental study, this factor can be considered in compensating the increase in cell survival due to the time prolongation.

It seems appropriate to use the method proposed by Mu *et al*. to predict the cell response following fractionation radiotherapy, especially in new fractionation radiotherapy procedures with more number of subfractions and with prolonged total treatment times. This method can simply and accurately determine the cell survival after each radiotherapy assessment and can be used to calculate the compensating dose for these treatment schedules. Although the effect of fractionation dose delivery is negligible for one session (with dose of 2 Gy), and it seems that there is no need to compensate these effect, but it can be important for a radiotherapy period (30 or 35 session with 2 Gy in each fraction) because of the cumulative effect of dose.

## Figures and Tables

**TABLE 1.** The F10B16 and 4T1 cell parameters as input data for the used models

**Symbols (unit)**	**Definitions**	**F10B16**	**4T1**
α (Gy^−1^)	Linear parameter of LQ model	0.0956	0.0424
β (Gy^−2^)	Quadratic parameter of LQ model	0.0177	0.0399
T_1/2_ (hour)	Half time of sublethal repair	0.524±0.035	0.344±0.015

**TABLE 2.** Experimental and calculated survival using method III for total dose of 4 Gy

		**Survival fractions of F10B16 and 4T1 cells**			

		**Experimental calculations**		**Theoretical calculations**	

**Number of subfractions×dose (Gy)**	**Total treatment time (min)**	**F10b16**	**4T1**	**F10b16**	**4T1**

**0×0**	**0**	**1**	**1**	**1**	**1**
1×4	5	0.518±0.019	0.459±0.017	0.513±0.038	0.445±0.012
2×2	15	0.535±0.027	0.506±0.018	0.534±0.036	0.505±0.048
2×2	30	0.549±0.017	0.547±0.018	0.550±0.023	0.545±0.019
2×2	60	0.570±0.016	0.588±0.017	0.569±0.029	0.587±0.042
2×2	120	0.586±0.016	0.609±0.018	0.585±0.019	0.609±0.027
2×2	180	0.590±0.018	0.612±0.019	0.590±0.054	0.612±0.038
2×2	240	0.591±0.029	0.613±0.016	0.591±0.024	0.613±0.029
4×1	30	0.546±0.026	0.546±0.017	0.542±0.038	0.529±0.035
8×0.5	30	0.549±0.015	0.547±0.026	0.540±0.034	0.523±0.043
4×1	60	0.570±0.017	0.585±0.017	0.567±0.047	0.595±0.047
8×0.5	60	0.570±0.016	0.583±0.008	0.564±0.028	0.589±0.038
4×1	240	0.598±0.018	0.653±0.027	0.622±0.024	0.706±0.038
8×0.5	240	0.607±0.008	0.674±0.008	0.627±0.023	0.732±0.042
